# Microelectrode Arrays for Simultaneous Electrophysiology and Advanced Optical Microscopy

**DOI:** 10.1002/advs.202004434

**Published:** 2021-05-11

**Authors:** Sagnik Middya, Vincenzo F. Curto, Ana Fernández‐Villegas, Miranda Robbins, Johannes Gurke, Emma J. M. Moonen, Gabriele S. Kaminski Schierle, George G. Malliaras

**Affiliations:** ^1^ Department of Chemical Engineering and Biotechnology University of Cambridge Cambridge CB3 0AS UK; ^2^ Electrical Engineering Division Department of Engineering University of Cambridge Cambridge CB3 0FF UK; ^3^ Department of Mechanical Engineering Microsystems Eindhoven University of Technology Eindhoven 5600 MB the Netherlands

**Keywords:** bioelectronics, conducting polymers, electrophysiology, optical microscopy

## Abstract

Advanced optical imaging techniques address important biological questions in neuroscience, where structures such as synapses are below the resolution limit of a conventional microscope. At the same time, microelectrode arrays (MEAs) are indispensable in understanding the language of neurons. Here, the authors show transparent MEAs capable of recording action potentials from neurons and compatible with advanced microscopy. The electrodes are made of the conducting polymer poly(3,4‐ethylenedioxythiophene) doped with polystyrene sulfonate (PEDOT:PSS) and are patterned by optical lithography, ensuring scalable fabrication with good control over device parameters. A thickness of 380 nm ensures low enough impedance and >75% transparency throughout the visible part of the spectrum making them suitable for artefact‐free recording in the presence of laser illumination. Using primary neuronal cells, the arrays record single units from multiple nearby sources with a signal‐to‐noise ratio of 7.7 (17.7 dB). Additionally, it is possible to perform calcium (Ca^2+^) imaging, a measure of neuronal activity, using the novel transparent electrodes. Different biomarkers are imaged through the electrodes using conventional and super‐resolution microscopy (SRM), showing no qualitative differences compared to glass substrates. These transparent MEAs pave the way for harnessing the synergy between the superior temporal resolution of electrophysiology and the selectivity and high spatial resolution of optical imaging.

## Graphical abstract

A transparent microelectrode array (MEA) technology based on the conducting polymer poly(3,4‐ethylenedioxythiophene) doped with polystyrene sulfonate (PEDOT:PSS) enables state‐of‐the‐art optical imaging along with electrophysiology. The electrodes maintain low noise under laser illumination and allow the detection of single unit activities from multiple putative neurons.

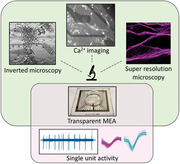

## 1 Introduction

The adoption of microelectrode array (MEA) technology in electrophysiology has played a pivotal role in supporting the study of electrogenic tissues both in vivo and in vitro.^[^ ^1^, ^2^ ^]^ When compared to patch clamp or microwire electrodes, the greater flexibility of MEAs in their design and use have made them the preferred option for the study of large cell populations. MEAs are capable of recording both low and high frequency signals arising from the fluctuation of ions across cell membranes. Implantable MEAs^[^ ^3^ ^]^ comprising microelectrodes made from metals and their compounds (e.g., Pt, Au, Ir, IrOx, TiN) find widespread applications for in vivo studies of the brain in animal models.^[^ ^3^, ^4^ ^]^ For in vitro applications, metal microelectrodes patterned on glass are extensively used to interface with cell cultures and tissue slices for drug screening and toxicology studies.^[^ ^1^, ^5^ ^]^ A recent trend in the field involves the use of conducting polymer electrodes. Materials such as poly(3,4‐ethylenedioxythiophene) doped with polystyrene sulfonate (PEDOT:PSS) have been successfully employed for the recording of neural activity in vivo^[^ ^6^, ^7^, ^8^ ^]^ and in vitro.^[^ ^9^, ^10^, ^11^ ^]^ PEDOT:PSS is a mixed electronic/ionic conductor with a capacitance that depends on the volume rather than the area of the film.^[^ ^12^ ^]^ Consequently, PEDOT:PSS electrodes have a significantly lower impedance compared to Pt and Au electrodes, which are limited by the capacitance of the electrochemical double layer formed at the metal/electrolyte interface. Their lower equivalent resistance in an electrolyte also reduces thermal noise, a major source of noise in electrical recordings.^[^ ^13^ ^]^ As a result, PEDOT:PSS electrodes lead to recordings with high signal‐to‐noise ratio (SNR) and to effective neural stimulation^[^ ^6^, ^9^, ^14^ ^]^ while being biocompatible and promoting neuron attachment and growth.^[^ ^15^ ^]^

In addition to electrophysiology, optical microscopy is an indispensable tool for neuroscience research due to its high spatial resolution and ability to target biomarkers selectively.^[^ ^16^, ^17^ ^]^ Fluorescence microscopy techniques are commonly used to highlight various structures within neurons and to visualize action potentials and network activity.^[^ ^18^, ^19^ ^]^ In addition to widefield microscopy, advanced techniques such as confocal microscopy boost resolution and contrast by collecting light selectively from a focal plane to reduce the impact of scattered light. Super‐resolution microscopy (SRM) techniques target biomarkers at the sub‐diffraction limit regime. Structured illumination microscopy (SIM), a popular SRM technique, is used for high‐speed imaging of neurons and the millisecond dynamics of action potentials. It works by acquiring separate images corresponding to phase shifted illuminations and computationally recovering higher spatial frequencies from individual frames.^[^ ^20^ ^]^ However, since most microelectrodes are based on metals, they are non‐transparent and incompatible with multiparametric imaging, for example, fluorescence lifetime imaging microscopy (FLIM)^[^ ^21^ ^]^ and also high resolution microscopy, which rely on inverted microscopes. In this context, optically transparent MEAs have received a great deal of attention over the recent years as a means to combine electrophysiology and optical imaging.

Transparent conductors, such as indium tin oxide (ITO) and graphene are probably the two most common materials employed for making transparent MEAs. ITO is widely used in optoelectronics applications and ITO‐based transparent MEAs are also commercially available. Single or multi‐layer graphene is attractive due to its >90% transparency window over the ultraviolet to infrared spectral range.^[^ ^22^, ^23^, ^24^, ^25^ ^]^ The impedance of ITO and graphene electrodes, however, is limited by the capacitance of the electrochemical double layer. Although PEDOT:PSS films are transparent, they have mostly been used as coatings over metal electrodes, leading to opaque MEAs. However, Au‐grid pattern,^[^ ^26^ ^]^ bilayer‐nanomesh of Au and conducting polymers,^[^ ^27^, ^28^ ^]^ carbon nanotube web‐like thin films,^[^ ^29^ ^]^ and bilayers of graphene and PEDOT:PSS^[^ ^30^ ^]^ have been used to make low‐impedance, transparent electrode, and contacts. By and large, these studies did not address electrode compatibility with advanced microscopy techniques. As the latter place significant demands on the geometry and optical properties of the substrate, it is not a priori clear that any transparent electrode would be suitable. Here, we report on transparent MEAs where the electrodes and the interconnects in their vicinity are exclusively made from a thin film of PEDOT:PSS. The thickness of the PEDOT:PSS film is optimized to navigate the trade‐off between low impedance and transparency. The transparent MEAs are shown to record action potentials from cortical neurons in vitro and support optical imaging of intracellular Ca^2+^ dynamics. Most importantly, laser illumination through the electrodes does not lead to light‐induced artefacts or additional recording noise and various fluorescently labelled molecular targets of different sizes are imaged through the MEA on inverted microscope setups. Moreover, the MEAs are shown to be compatible with SIM, a high‐speed super‐resolution technique, with negligible effect on image resolution.

## 2 Results and Discussion

**Figure 1:**
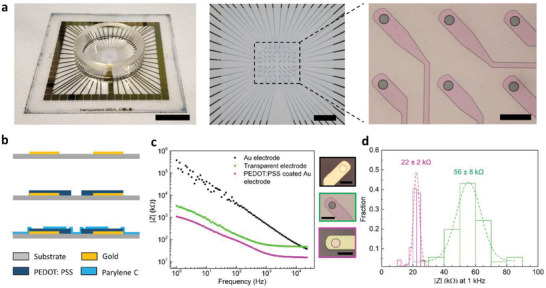
Images of the MEAs and electrochemical performance. a) Optical image of the transparent MEA (left), close‐up view of the transparent recording region (middle), and optical micrograph of the recording electrodes (right). Scale bars: left, middle 1 cm; right, 100 µm. b) Schematic illustration of the fabrication process of the PEDOT:PSS transparent electrodes. c) Comparison of the electrochemical impedance spectra of representative electrodes from MEAs with PEDOT:PSS (green), Au (black), and PEDOT:PSS‐coated Au (magenta)electrodes. Optical micrographs of each type of electrodes are shown in the inset. Scale bar: 50 µm. d) Distribution of the impedances of the PEDOT:PSS (green, *n* = 42) and PEDOT:PSS‐coated Au electrodes (magenta, *n* = 47) at 1 kHz. The dotted lines represent Gaussian fits to the histograms.

In order to ensure compatibility with commercial microscope lenses that have high magnification and high numerical aperture (NA), the MEAs were fabricated on ≈170 µm thick glass substrates which correspond to #1.5 coverslips. **Figure** **1**a shows pictures of the whole device with an attached cell culture well (left), the electrode recording area (middle), and six recording electrodes (right). The transparent optical window located at the center of the cell culture well has a total surface area of approximately 4×4 mm^2^. Within this region, 60 recording sites are located on an 8×8 square grid, with a center‐to‐center distance of 200 µm. Each recording site has a diameter of 30 µm (area of ≈700 µm^2^). The recording sites are connected with PEDOT:PSS and Au interconnects to the peripheral contact pads of the MEA. Au interconnects are used outside the transparent window to minimize resistive losses. The layout of the contact pads was designed to be compatible with a commercially available in vitro recording system (MEA2100 mini headstage, Multichannel Systems MCS GmbH, Germany).

Figure 1b illustrates the main fabrication steps (more details included in the Experimental Section below). Au pads/interconnects are first patterned on glass, followed by the deposition and patterning of the PEDOT:PSS electrodes. The latter is performed by dry etching of PEDOT:PSS using a commercial photoresist as an etch mask. A critical step in this fabrication process is the overnight soaking of the deposited PEDOT:PSS film in de‐ionized (DI) water to remove the surface PSS layer that would otherwise interfere with the photoacid chemistry of the photoresist.^[^ ^31^ ^]^ We also attempted fabrication using PEDOT:PSS lift‐off in organic solvents or peel‐off of a sacrificial Parylene C (PaC) layer. These techniques often resulted in defects/breaks in the PEDOT:PSS film (see Figure S1, Supporting Information). Surface modification of the Au interconnects by a self‐assembled monolayer (SAM) of 3‐mercaptopropyltrimethoxysilane (MPTMS) improved adhesion, but we found that etching the PEDOT:PSS yielded more consistent results. The fabrication of the MEAs was completed with the deposition of an ≈500 nm thick insulating layer of PaC that was patterned to expose the underlying PEDOT:PSS recording site and Au contact pads.

The performance of the transparent MEAs was compared to that of devices with Au electrodes of the same size, as well as devices with PEDOT:PSS‐coated Au electrodes. Optical micrographs of these electrodes are shown in Figure S2, Supporting Information. The Au film thickness was 100 nm, making the central window of these MEAs non‐transparent. The PEDOT:PSS film had a thickness of ≈380 nm, selected to optimize the impedance versus transparency trade‐off (see below). The electrochemical impedance spectra (magnitude plot) of the three different electrode designs are shown in Figure 1c. The Au electrodes exhibited the highest impedance, around 10 times higher than that of the transparent electrodes at 1 kHz, the frequency that is relevant for detecting action potentials. On the other hand, since Au has lower sheet resistance (0.24 Ω sq^−1^) than PEDOT:PSS (86 Ω sq^−1^), the presence of the Au underlayer in the PEDOT:PSS‐coated Au electrodes reduces their overall resistance. While the Au interconnects have a mean resistance of 76.5 Ω, it was 6.5 kΩ for the transparent PEDOT:PSS interconnects. The high frequency values of impedance in Figure 1c reflect this difference in resistance. This ultimately results in an ≈2.5 times lower impedance at 1 kHz, compared to the transparent PEDOT:PSS electrodes. The phase plots of the three different types of electrodes are shown in Figure S3, Supporting Information. The histogram in Figure 1d illustrates the distribution of impedance values of the PEDOT:PSS and PEDOT:PSS‐coated Au electrodes at 1 kHz. The data include electrodes made in different batches of fabrication. A gaussian fit reveals an average impedance of ≈56 ± 8 kΩ (mean ± standard deviation, SD) for the PEDOT:PSS electrodes, and ≈22 ± 2 kΩ for the PEDOT:PSS‐coated Au electrodes. Different batches did not show statistically significant variation in impedance, as inferred from one‐way analysis of variance (ANOVA) tests (p = 0.29, F = 1.17, Figure S4, Supporting Information). A comparison with the impedance of other transparent MEAs reported in literature is presented in Table S1, Supporting Information. It should be noted that the typical yield of functional PEDOT:PSS electrodes per MEA was 95%.

**Figure 2:**
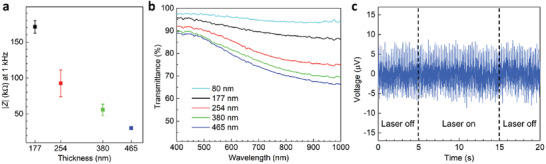
Impedance versus transparency trade‐off and noise performance. a) Comparison of average impedances (at 1 kHz) of transparent electrodes fabricated from PEDOT:PSS films of different thickness (*n* = 3). b) Optical transmittance of PEDOT:PSS films of different thickness (*n* = 3). c) Effect of scanning a confocal laser beam on the electrical recordings from a transparent electrode immersed in PBS. The laser was scanned between 5 and 10s from the start, shown by the dashed lines.

It is known that thicker PEDOT:PSS films lead to lower impedance,^[^ ^12^ ^]^ which is desirable for higher SNR in electrophysiology recordings. This is shown in **Figure** **2**a where the impedance at 1 kHz decreases from ≈170 kΩ for 177 nm thick electrodes to ≈30 kΩ for 465 nm thick electrodes (see also Figure S5a, Supporting Information). The lower sheet resistance of the thicker PEDOT:PSS films (see Figure S5b, Supporting Information) also reduces the resistance of the resulting interconnects. However, thicker electrodes are also less transparent. **Figure** 2b compares the optical transmittance through PEDOT:PSS thin films of thickness varying between 80 and 465 nm. We selected ≈380 nm as the optimum film thickness for further investigation, as it ensures >75% transparency across the visible part of the spectrum and shows a relatively low impedance.

**Figure 3:**
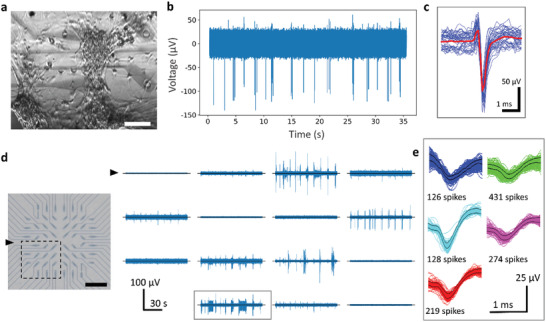
Electrophysiology and spike classification. a) Brightfield image of primary neurons on the transparent MEA. Scale bar: 50 µm. b) Spontaneous activity of primary neurons recorded from a transparent electrode. c) Aggregated waveform of spikes detected from the recording in (b) aligned to their negative peak amplitudes (shown in blue). Red line shows their average. d) High pass filtered recording traces showing spontaneous activity from adjacent electrodes of the transparent MEA (right). The location of the electrodes is highlighted on the electrode‐map of the MEA (left). The arrow indicates the reference electrode. Scale bar: 1mm (left). e) Classification of the spikes detected at the highlighted electrode in (d) depending on their shapes. Different groups are shown in different colors. The black outline represents the boundary of a spike template which is overlaid with individual spike waveforms.

The MEAs were subsequently validated in vitro using primary cortical cell cultures. A first experiment was carried out to verify that shining a laser through the electrodes did not increase the recorded electrical noise. Figure 2c shows a recording in phosphate buffered saline (PBS) solution (without neurons), with and without illumination by the 480 nm laser of a confocal microscope. Shining the laser though the electrodes does not increase noise, which stays within 20 µV (peak to peak, 2.44 µV rms). Next, we demonstrated recordings from cortical neurons. The preparation and maintenance of the cell cultures are described in the Experimental Section. **Figure** **3**a illustrates brightfield image of the MEAs with neurons (days in vitro, DIV 23), acquired by an inverted optical microscope. Under visual inspection, the neurons appeared viable on the PaC insulation layer, and they formed a well interconnected network of neuronal processes. The neuronal cell bodies and bundles of axons can be clearly observed through the electrodes. Figure 3b shows an instance of raw (unfiltered) recordings of spontaneous activity from an electrode at 21 DIV. In terms of the electrophysiology recordings, we focused on action potentials since these form the basis of communication in neurons. Hence, the wideband signal recorded from the MEA was digitally filtered using a high pass filter (cut‐off frequency 200 Hz) to reject the low frequency oscillations as well as the 50 Hz power line noise. The spiking events were detected by setting a threshold (5 x SD) and the spike waveforms were collapsed over a 4 ms window centered at the time of the peaks, for computing the average. The SNR of the raw signal, calculated from the mean spike amplitude and the SD of the background, was found to be 7.7 (17.7 dB). Figure 3c shows a limited number of detected spike waveforms (blue) overlaid with their average (red). At cell densities of 700 per mm^2^ and 900 per mm^2^, significant neuronal activity could be observed in 16 out of 60 electrodes (59 recording electrodes and 1 reference electrode) on an average (both cell densities, 5 recording sessions). Figure 3d (right) shows the high pass filtered recording traces obtained from several adjacent electrodes of the MEA during a recording session. The 15 electrodes correspond to a quadrant of the MEA, as highlighted on the left image. The action potential spikes detected in an electrode recording can originate from more than one nearby neuron. Their amplitudes vary depending on their location relative to the electrode, which explains different heights of the spikes observed in any recordings of Figure 3d. Apart from their amplitudes, different neurons are expected to have different spike shapes. The low background noise of the recordings facilitated the classification of spikes into different groups by a method of template matching. In this method templates were created from initially observed spikes and the subsequent spikes were matched with the closest template (see Experimental Section for details). A time window of 1.4 ms was chosen to reject wider spikes resulting from superposition of multiple action potentials. Only the recordings with a high enough number of spikes are analyzed and, in each case, multiple spike shapes are seen which may correspond to putative neurons. For example, the highlighted recording in Figure 3d consists of five major categories of spikes, as shown by different colours in Figure 2e. The black lines represent the boundaries of the templates calculated from the matched waveforms. Figure S6, Supporting Information shows the spike groups from the other electrodes.

**Figure 4:**
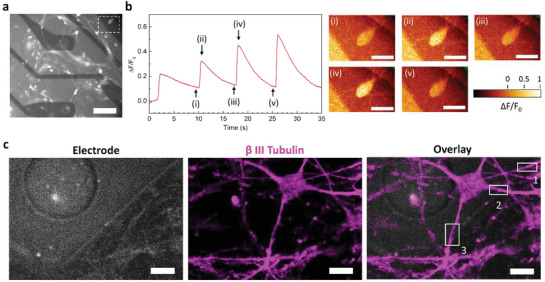
Ca^2+^ imaging and confocal microscopy. a) Fluorescence microscopy image of neurons labelled with Ca^2+^ indicator dye Fluo‐4, on the transparent electrodes. Scale bar: 50 µm. The dotted box highlights a neuron located on a PEDOT:PSS interconnect. b) Left: Normalised variation of fluorescence intensity (*ΔF/F*0) over time for the neuron cell body highlighted in (a). Right: False coloured fluorescence images of the neuron at different times marked in the left plot. Scale bar: 5 µm. c) Confocal microscopy images of neurons chemically fixed on the MEA and immuno‐labelled for the cytoskeleton marker *β*‐III‐tubulin (magenta). The electrode can be faintly seen in the background. The numbered boxes indicate regions where the neuronal processes cross the electrode boundary. Scale bar: 5 µm.

In addition to electrophysiology recordings, the transparency of the PEDOT:PSS electrodes enabled Ca^2+^ imaging of neurons using an inverted fluorescence microscope. Given the importance of Ca^2+^ ions as intracellular messengers in neurons, Ca^2+^ imaging has been an essential tool in neuroscience over the past decades. In this method, the fluorescence of externally introduced Ca^2+^ binding dyes or genetically encoded calcium indicators are used to determine the dynamics of intracellular Ca^2+^ ion concentration. Here, a high affinity Ca^2+^ indicator Fluo‐4 was used, whose fluorescence intensity (emission wavelength = 520 nm) can be correlated to neuronal activity. **Figure** **4**a shows Fluo‐4 labelled neurons on the transparent electrodes and the corresponding video is presented in Video S1, Supporting Information. The glowing cell bodies and neurites indicate the spontaneous activity of the neuronal network. The presence of PEDOT:PSS did not have any observable effect on Ca^2+^ fluorescence. The dotted box in Figure 4a highlights a highly active neuron located on a PEDOT:PSS interconnect. The normalized fluorescence intensity variation (*ΔF/F_0_*) of the cell body over time is illustrated in the left plot of Figure 4b. The prominent changes in the *ΔF/F_0_* during two consecutive spikes in Ca^2+^ fluorescence can also be visualized from the images on the right. Each spike is characterized by a very sharp rise (<100 ms) in intensity followed by a gradual decay to the base fluorescence lasting for 7–8 s.

The stability of the PEDOT:PSS electrodes was evaluated by measuring their impedances before and after 21 days of cell culture. A comparison of the electrochemical impedance spectra in Figure S7, Supporting Information, shows that, on an average, the impedance reduced marginally across the whole frequency range. This can be due to the enlargement of the recording sites owing to the swelling of PEDOT:PSS. Previously, our group has also shown the long‐term stability of PEDOT:PSS coated Au electrodes in cell culture conditions.^[^ ^32^ ^]^

Along with live fluorescence imaging, the MEAs were further validated with widefield and confocal microscopy on fixed neuronal cultures. Figure S8a, Supporting Information illustrates the widefield images of neurons stained for *β*‐III‐tubulin (left), bassoon protein (middle), and overlap of both channels (right). The circular electrode is also highlighted for clarity. *β*‐III‐tubulin is almost exclusively found in neurons and is a common marker for the cytoskeleton in neurons. Bassoon is a structural protein present in the presynaptic active zone, the region where neurotransmitters are released by fusion of synaptic vesicles with the nuclear membrane. The large difference in the sizes of these particular biomarkers made them an apt model for validating the transparency, and impacts on the optical resolution when imaging through the PEDOT:PSS electrodes. The appearance of the tubulin cytoskeleton across the electrode was very similar to that of neurons fixed on a conventional glass substrate. This similarity was expected since the cytoskeleton is a large and continuous structure, which is less likely to be perturbed by the PEDOT:PSS layer. On the contrary, the bassoon proteins were more challenging as they occur as discrete nanometer‐sized puncta that are closely interspaced. Interestingly, they could be clearly observed through the electrode in Figure S8a, Supporting Information. Figure S8b, Supporting Information, presents a clearer view of an isolated neurite located on the transparent electrode where the bassoon puncta (green) are observed along the continuous neurite projection.

Figure 4c illustrates the confocal images of neurons chemically fixed on the MEA and stained for *β*‐III‐tubulin (middle). The electrode plane could be observed at 488 nm illumination (left). Closer inspection of the overlaid image (right) reveals that the neuronal processes appear brighter outside the electrode compared to when they are located on it. Three instances of such differences in fluorescence intensity have been highlighted (Figure 4c, numbered boxes). Figure S9, Supporting Information shows that the average pixel intensities of regions located on the transparent electrode was ≈70% of the substrate. However, the electrode did not create any qualitative differences to the image.

**Figure 5:**
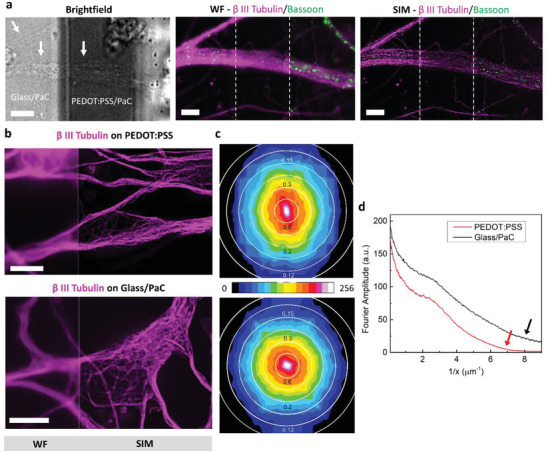
Structured illumination microscopy through the transparent MEAs. a) Brightfield image of neurites (arrows) passing over PEDOT:PSS interconnects (left). Widefield image (middle) and reconstructed SIM image (right) of the same image on the left, labelled for *β*‐III‐tubulin (magenta) and Bassoon (green). The dashed lines denote the underlying PEDOT:PSS layer. Scale bar: 5 µm. b) Widefield‐SIM images of the microtubule networks in neurons located on PaC insulated PEDOT:PSS electrode (PEDOT:PSS/PaC, top) and on the glass surface of the MEA (glass/PaC, bottom). Scale bar: 5 µm. c) Fourier amplitude versus spatial frequency heatmaps obtained from 2D Fourier transforms of the respective reconstructed images in (b). The concentric rings denote the feature sizes (in µm) of the image corresponding to the spatial frequencies. The Fourier amplitudes range from 0 to 256 as depicted in the color bar. d) Radial plots of circularly averaged Fourier amplitudes for the 2D Fourier transformations in (c). The arrows indicate the inflection points in the curves which approximate the resolution limit.

Finally, we demonstrated that the PEDOT:PSS electrodes are compatible with SIM. The brightfield image in **Figure** **5**a left illustrates neurites (arrows) on a transparent PEDOT:PSS interconnect. The corresponding widefield (middle) and reconstructed SIM images (right) show the cytoskeleton (*β*‐III‐tubulin, magenta) and a presynaptic marker (bassoon, green). The location of the PEDOT:PSS interconnect is highlighted by dotted lines. Individual microtubules can be observed in the reconstructed SIM image which are otherwise absent in the widefield image. The bassoon puncta are significantly better resolved compared to widefield images in Figure S8, Supporting Information, with an average size of 240 nm. It can be observed that the underlying PEDOT:PSS layer has a minor effect on the pixel intensity which stems from the difference in focus due to its thickness. The improvement in resolution, is better captured in the 2D Fourier transform analysis which measures the spatial frequency information present in an image. Figure S10a, Supporting Information, shows the 2D Fourier transforms of the widefield and SIM images (Figure 5a) of the bassoon marker. Please note, the averaged radial plots in Figure S10b, Supporting Information, illustrate a much higher resolution in the SIM image.

Figure 5b shows the widefield‐SIM images of microtubules in the cell body and neurites located on PEDOT:PSS/PaC (top) and glass/PaC (bottom). The image reconstructions are also compared quantitatively from the pixel intensity histograms for both images (refer to Experimental Section). Figure S11, Supporting Information, illustrates the mode intensity and the portion of fluorescence intensities on either side of it. The min‐to‐max ratio (MMR) calculated from the histogram gives the intensity of useful features relative to the reconstructed noise. The MMRs were found to be 15 and 4.6 for the PEDOT/PaC and glass/PaC surfaces respectively, indicating a better reconstruction in the former case. However, intensity‐based measures like MMR have the limitation that they depend on the concentration of the fluorescent marker and the fraction of image area they cover. The 2D Fourier transform heatmaps shown in Figure 5c (greyscale image in Figure S12, Supporting Information) correspond to the SIM images in Figure 5b and are a suitable alternative for qualitative comparison. The skewed nature of the heatmaps indicates different resolution along different directions in the image. Similar observation for both surfaces (PEDOT:PSS/PaC as well as glass/PaC) suggests that it is caused by common parameters, one of which could be the illumination. Gradually decaying Fourier amplitudes with increasing spatial frequency indicate useful high frequency information in the image, while a plateau‐like profile implies noise.^[^ ^33^ ^]^ Figure 5d compares the radial profiles of averaged Fourier amplitudes obtained from the Fourier transforms in Figure 5c. The resolution limit of an image is approximated by the inflection point in the radial profile beyond which noise is predominant. The slightly higher curvature in the PEDOT:PSS/PaC profile around *x* = 7 (red arrow), that is, 140 nm can be attributed to marginally higher noise compared to the glass/PaC surface, where the limit is around *x* = 8 (black arrow), that is, 125 nm. Thus, light absorption by the underlying PEDOT:PSS film (≈77% transmittance at 647 nm, the emission wavelength of the cytoskeleton label) does not affect imaging by SIM.

## 3 Conclusion

The transparent MEAs presented here are compatible with both high SNR electrophysiology recordings and advanced microscopy techniques. The PEDOT:PSS electrodes were optimized to achieve >75% transparency and low electrochemical impedance. The latter was enabled by the volumetric capacitance of PEDOT:PSS, which represents a major advantage compared to other transparent electrodes including ITO and graphene. Their patterning with conventional lithography ensures a scalable process, capable of delivering a valuable tool for neuroscience at scale.

## 4 Experimental Section

### Materials

The PEDOT:PSS water dispersion (Clevios PH 1000, Heraeus, Germany) was chemically modified with 5% (v/v) ethylene glycol and ≈30 µL of dodecyl benzene sulfonic acid (DBSA). The mixture was sonicated and 1% (v/v) 3‐glycidyloxypropyl)trimethoxysilane (GOPS) was added just before use. A polytetrafluoroethylene (PTFE) filter (0.45 µm pore size) was used to filter the mixture.

### Fabrication

Initially ≈5×5 cm^2^ glass substrates (170 µm thick) were cleaned in soap solution followed by rinsing in DI water, acetone, and isopropyl alcohol. After dehydration baking, they were spin coated with negative lift‐off photoresist AZnLOF 2035 (Microchemicals GmbH, Germany) and UV exposed using a mask aligner (MA/BA 6, Suss MicroTec, Germany). Au contacts were defined by electron beam evaporation of Ti (5 nm) and Au (100 nm) and subsequent lift off. In samples where lift‐off of PEDOT:PSS was attempted, a SAM of MPTMS was deposited on Au as described in literature^[^ ^34^ ^]^ to enhance PEDOT:PSS adhesion (refer to Figure S1, Supporting Information). The PEDOT:PSS formulation was spin coated and the film was baked for 1 h at 110 °C and soaked overnight in DI water in order to remove excess PSS and low molecular weight compounds. A photoresist etch mask (≈2.2 µm) was lithographically defined with the photoresist AZ 5214E (Microchemicals GmbH, Germany). The PEDOT: PSS layer was subsequently etched by reactive ion etching using CF_4_ and O_2_ (5 sccm and 50 sccm flowrates respectively, at 60 mTorr pressure and 150 W power). After rinsing of the remaining photoresist with acetone, a SAM of the adhesion promoter methacryloxypropyl trimethoxysilane (A 174 Silane, Sigma‐Aldrich, UK) was created to improve the adhesion of the PaC insulation layer to the glass substrate. The silanization was done by dipping the plasma activated (60 s, 25 W, 0.8 mbar) substrate in 3% (v/v) A 174 solution prepared in 96% ethanol (containing 1% acetic acid) for 30 s, rinsing off with ethanol, and baking at 70 °C for 1 h. ≈500 nm thick PaC layer was deposited by chemical vapor deposition (SCS Labcoater, Speciality Coating Systems, US). This was followed by patterning of AZnLOF 2035 as the etch mask to define the openings of electrodes and Au contact pads (in the periphery of the glass substrate) through the PaC layer. The etch rate of PaC was determined in prior experiments and was used to control its etching to avoid oxidation and damage to the underlying PEDOT: PSS layer. Residues of the etch mask were removed by rinsing with copious amount of acetone, followed by rinsing with iso‐propyl alcohol and DI water. The Au and PEDOT:PSS‐coated Au electrodes were fabricated in a similar manner as described above.

### Optical Characterization

For UV–vis spectrometry, a fiber optic spectrometer (AvaSpec‐ULS2048CL‐EVO, Avantes), coupled to a deuterium halogen light source (AvaLight‐DH‐S, Avantes), and a temperature controlled sample holder. The measurements were conducted at 25 °C. The PEDOT:PSS samples on glass were processed as described above. The film thickness was verified via a Stylus Profilometry (Dektak XT, Bruker).

### Electrical Characterization

The impedance of the electrodes was characterized by an Electrochemical Impedance Spectroscopy (Autolab Potentiostat, Metrohm AG, Switzerland) with a platinum electrode as the counter electrode. A sinusoidal voltage input of amplitude 10 mV at different frequencies ranging from 1 to 100 kHz was used for the purpose.

### Preparing MEAs for Cell Plating

Prior to processing the MEAs for cell cultures, the impedances of a few electrodes for each MEA were measured in PBS. Since the SNR of the recorded signal is decided by impedance, this test determined whether the MEA is suitable for electrophysiology recordings. MEAs with average impedances less than 100 kΩ were accepted for electrophysiology. The MEAs were prepared for cell cultures in a similar process as reported earlier.^[^ ^11^ ^]^ Concisely, the MEAs were treated with mild O_2_ plasma (60 s, 25 W, 0.8 mbar) and kept soaked in DI water to make the PaC surface hydrophilic. The MEAs were subsequently sterilized by 70% ethanol (for 30 min) and rinsed with Dulbecco's phosphate buffered saline (DPBS, Thermo Fischer Scientific, UK). Next, a coating of poly‐L‐lysine (PLL, 0.005 wt% in PBS, Sigma‐Aldrich, UK) was applied for 2 h. The MEAs were rinsed with DPBS; the cell culture medium was added and kept in the incubator until plating cells.

### Primary Cell Culture

Cortical tissues were isolated from postnatal day 1 rats (Sprague–Dawley rats from Charles River) and digested in Dulbecco's modified eagle medium (DMEM, Thermo Fischer Scientific, UK) containing 0.1% Trypsin and 0.05% DNAase (Sigma–Aldrich, UK) for 20 min in an incubator. The tissues were dissociated to single cell suspension by trituration through 1 mL and 200 µL Gilson pipette tips and the suspension was centrifuged at 600 rpm for 5 min. The supernatant was replaced by Neurobasal medium conaining 2% B27 and 0.25% Glutamax (all from Thermo Fischer Scientific, UK) and the cell pellet was gently resuspended. Finally, cells were plated on the MEAs containing the culture medium (NbActiv 4, BrainBits LLC, USA) at a cell density of 700–900 cells per mm^2^. The cultures were maintained by replacing half of the medium every 3 days.

### Electrophysiology and Spike Classification

Electrical recording in neuronal cell cultures was performed by an MEA2100 mini headstage and amplifier from Multichannel Systems MCS GmbH. All recordings were carried out at 21–23 DIV. Shape based spike classification was performed by a template matching method implemented in the Spike 2 software (Cambridge Electronic Design Limited, UK). First, the threshold was set for each recording channel, usually at ≈5 x SD, and a time window of 1.4 ms was selected. Spike templates with a tolerance of 20% were automatically created from the initial spikes. Later spikes were compared with each template and assigned to the closest match where at least 60% of the datapoints fell within the template bounds. New templates were considered only when similar shapes occurred at least once in 50 consecutive spikes.

### Ca^2+^ Imaging of Neurons

Ca^2+^ imaging of neurons was performed by a cell permeable fluorescent Ca^2+^ indicator dye, Fluo‐4 AM (Invitrogen, ThermoFisher Scientific, UK). The dye was introduced in the cell medium at a final concentration of 2 µm in the presence of 0.02% Pluronic F‐127 (Sigma–Aldrich, UK) surfactant. The neurons were incubated for 40 min to 1 h, in an incubator before imaging. An inverted fluorescence microscope (Olympus IX 71) with LED illumination at 488 nm and emission filter at 520 nm was used for the imaging. Images were acquired through a 40x air objective, with a CMOS camera (ZYLA 4.2, Andor, Oxford Instruments) at 50 frames per second, for 40–60 s. At the end of the imaging session, most of the dye containing medium was replaced with fresh medium. The image analysis was performed in ImageJ.^[^ ^35^ ^]^ The effect of bleaching was corrected by fitting a baseline of exponential decay and subtracting it from the measured florescence intensity. For individual neurons, the average intensity over the cell body was considered. The normalized variation in fluorescence intensity (*ΔF/F*_0_) was calculated as:

(1) $$\Delta F/F_{0}=\frac{F-F_{0}}{F_{0}}\;$$

Here, *F* and *F*_0_ denote the fluorescence intensities at an instant and during the neuron's resting state, respectively.

### Immunohistochemistry for Fluorescent Imaging

For microscopy, the cultured cells were fixed on the MEAs with 4% paraformaldehyde containing 0.12 m sucrose for 10 min. The cells were washed with PBS and incubated in the blocking buffer (PBS with 5% donkey serum and 0.05% Tween; Sigma–Aldrich, UK) for 1 h. The primary rabbit anti *β*‐III‐tubulin (1:400) and mouse anti bassoon (1:800) antibodies were subsequently added, and 1 h incubation time was allowed. Subsequently, it was washed three times with blocking buffer and the secondary donkey anti rabbit Alexa Fluor 647 and goat anti mouse Alexa Fluor 568 antibodies (both 1:400) were added for 10 min. After the final washing, PBS‐azide was added and the sample stored at 4 °C. All antibodies were procured from Abcam, UK.

### Optical Microscopy

SIM imaging was performed using a custom system built around Olympus IX 71 microscope.^[^ ^36^ ^]^ It was used for widefield imaging as well. The three‐color system consisted of laser wavelengths of 488 nm (iBEAM‐SMART‐488, Toptica), 561 nm (OBIS 561, Coherent), and 640 nm (MLD 640, Cobolt). A ferroelectric binary spatial light modulator (SXGA‐3DM, Forth Dimension Displays) patterned the light and the polarization was controlled with a Pockels cell (M350‐80‐01, Conoptics). A 60x/1.2NA water immersion lens (UPLSAPO 60XW, Olympus) focused patterned light on the substrate and also captured the fluorescent emission for projecting onto an sCMOS camera (C11440, Hamamatsu). The acquisition was done with the HCImage software (Hamamatsu) and customised LabView (National Instruments, USA) program.

### Image Analysis and SIM Reconstruction

Intensity analysis of confocal images was performed in ImageJ.^[^ ^35^ ^]^ The raw SIM images were reconstructed using a custom script that provided an interface to the fairSIM plugin^[^ ^37^ ^]^ in ImageJ. Comparative analysis of reconstructed images was carried out with the SIMcheck plugin^[^ ^33^ ^]^ as follows: The MMR metric was calculated as the ratio of the averaged 0.001% highest (Max*) and lowest (Min*) intensity pixels relative to the mode:

(2) $$MMR=\frac{Max^{*}-Mode}{\left|Min^{*}-Mode\right|}$$

2D Fourier transform was calculated similar to ImageJ's default fast fourier transform (FFT) method and the output was given as 8‐bit log(Amplitude^2^) which was finally expressed as heatmap with 16‐color look up table (LUT).

## Conflict of Interest

The authors declare no conflict of interest.

## Supporting information

Supporting Information

Supporting Information

## Data Availability

Data available on request from the authors
