# Supplementary material for: Microelectrode Arrays for Simultaneous Electrophysiology and Advanced Optical Microscopy
Source: Adv Sci (Weinh). 2021 May 11;8(13):2004434. doi: 10.1002/advs.202004434 (PMC9539726; doi:10.1002/advs.202004434)
Supplement: Supplementary file 1 — Supporting Information [file ADVS-8-2004434-s001.pdf]

© 2021 Wiley-VCH GmbH

ADVANCED SCIENCE
Open Access

Supporting Information

for *Adv. Sci.,* DOI: 10.1002/advs.202004434

Microelectrode arrays for simultaneous electrophysiology and advanced optical microscopy

Sagnik Middya , Vincenzo F. Curto , Ana Fernández-Villegas , Miranda Robbins , Johannes Gurke, Emma J. M. Moonen, Gabriele S. Kaminski Schierle, and George G. Malliaras **

Supporting Information

Microelectrode arrays for simultaneous electrophysiology and advanced optical microscopy

Sagnik Middya [1,2§], *Vincenzo F. Curto* [2§], *Ana Fernández-Villegas* [1], *Miranda Robbins* [1], *Johannes Gurke* [2], *Emma J. M. Moonen* [2,3], *Gabriele S. Kaminski Schierle* [1,*], *and George G. Malliaras* [2,*]

([§]shared first author)

[1] Department of Chemical Engineering and Biotechnology, University of Cambridge, CB3 0AS, UK
[2] Electrical Engineering Division, Department of Engineering, University of Cambridge, CB3 0FF, UK
[3] Department of Mechanical Engineering, Microsystems, Eindhoven University of Technology, 5600MB Eindhoven, Netherlands

* corresponding authors. E-mail: gsk20@cam.ac.uk, gm603@cam.ac.uk,

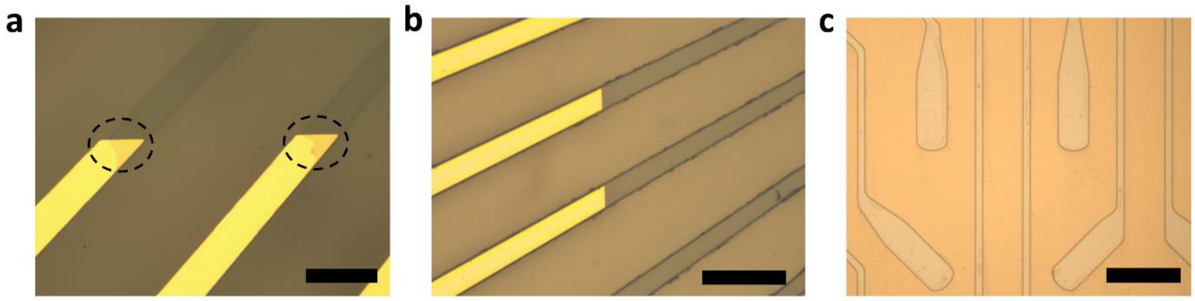

Figure S1. (a) PEDOT:PSS patterned by lift-off without surface modification of Au. The encircled regions show the poor adhesion between Au and the polymer where they come in contact. Scale: 100 µm. (b) Better adhesion between Au and PEDOT:PSS after surface modification of Au lines with self-assembled monolayer of 3-mercaptopropyltrimethoxysilane. Scale: 200 µm. (c) Transparent electrodes patterned by etching PEDOT:PSS. Scale: 100 µm.

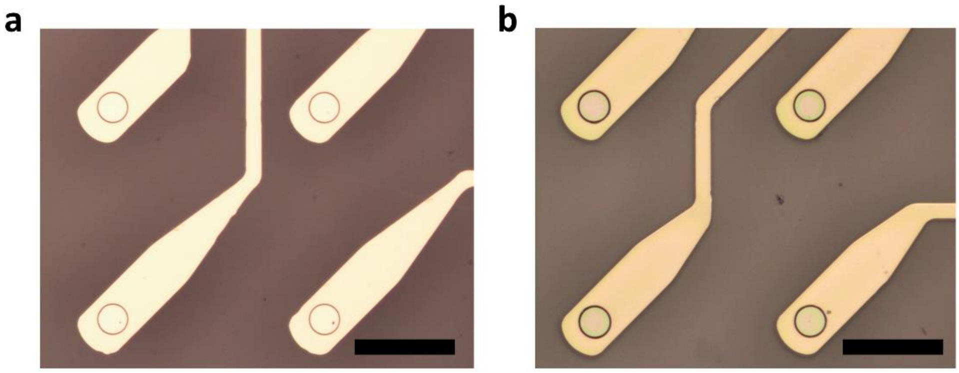

Figure S2. Optical micrographs of non-transparent (a) Au electrodes and (b) PEDOT:PSS-coated Au electrodes. Scale: 100 µm

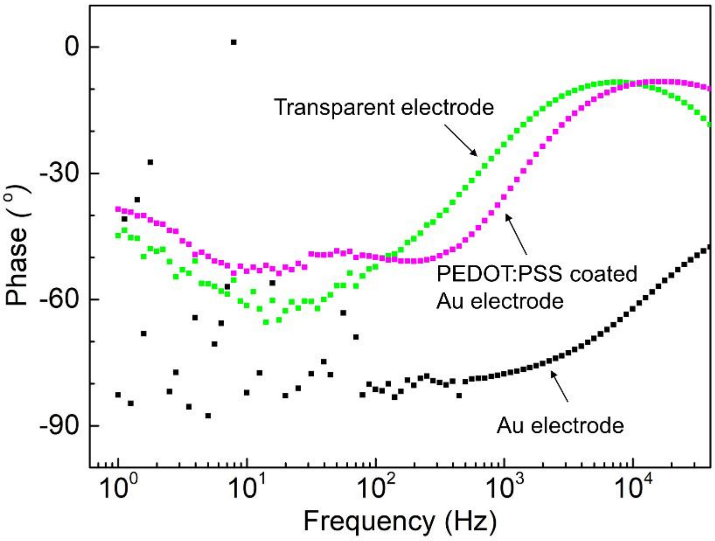

Figure S3. Phase plots of Au, PEODT:PSS coated Au and transparent electrode corresponding to the magnitude plots of the electrochemical impedance spectra presented in Figure 1c.

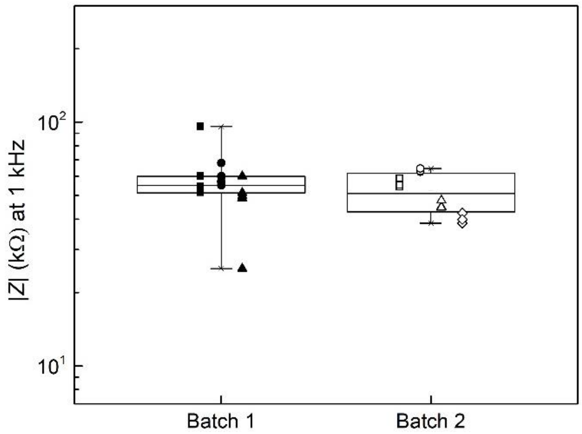

Figure S4. Variation of impedances of transparent electrodes from two batches of MEA fabrication. Here, each data point refers to an individual electrode, and similar shapes refer to the same MEA device (n = 15, across 3 MEAs of batch 1; n = 12, across 4 MEAs of batch 2) . No statistically significant difference in the population means of the batches were observed ($p > 0.05$, $F = 1.17$). Table S1. Comparison of previously reported transparent electrodes with the PEDOT:PSS transparent electrodes reported in the present work.

Material	Area-specific impedance (Z' = ZA), at 1 kHz (Ω cm^2)	Reference
ITO	2.2 – 6.28	[1]
ITO	8.95 – 35.81	[2]
Graphene	13.52	[3]
Graphene	~76.45	[4]
Au nanomesh	10.02	[5]
Au/PEDOT bilayer nanomesh	0.41 – 0.83	[6]
Graphene-PEDOT: PSS bilayer	0.29 – 1.17	[7]

**PEDOT: PSS**	**0.39**	**This work**

The transparent electrodes in Table S1 are compared in terms of their area-specific impedance, calculated as Z’ = ZA, where Z and A denote the measured impedance (in Ω) and electrode area (in cm^2) respectively. It provides a normalised impedance for an electrode with respect to its area.

When it comes to the bilayer design, gold electrodes coated with similar thickness of PEDOT:PSS through peeling off a sacrificial Parylene C layer have shown ~ 23 kΩ impedance for a 400 μm^2 electrode area.[8] The ~ 22 kΩ impedance of the bilayer electrodes (~700 μm^2 electrode area) reported here, is comparatively higher. This difference can be attributed to the exposure of the PEDOT:PSS surface to photoresist and solvents. However, as mentioned earlier, dry etching was found to be more convenient and reliable for patterning transparent electrodes as opposed to using a sacrificial Parylene C layer.

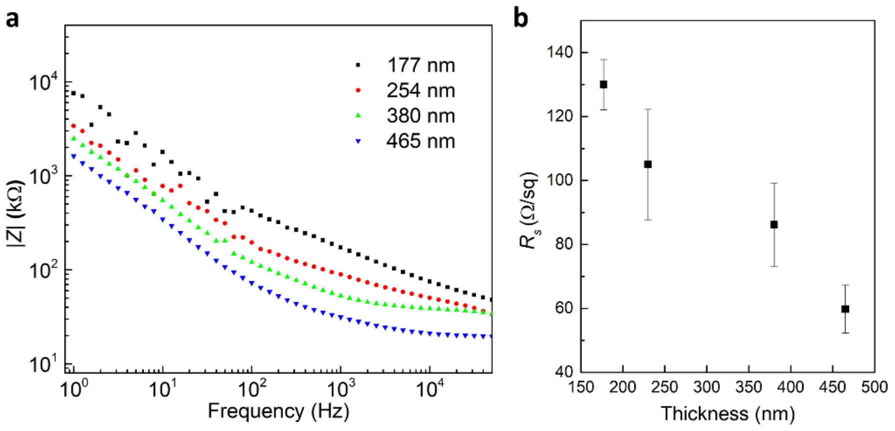

Figure S5. (a) Impedance spectra of transparent electrodes made from PEDOT:PSS films with differing thicknesses. (b) Variation of the sheet resistances (R_S) of PEDOT:PSS films with their thicknesses.

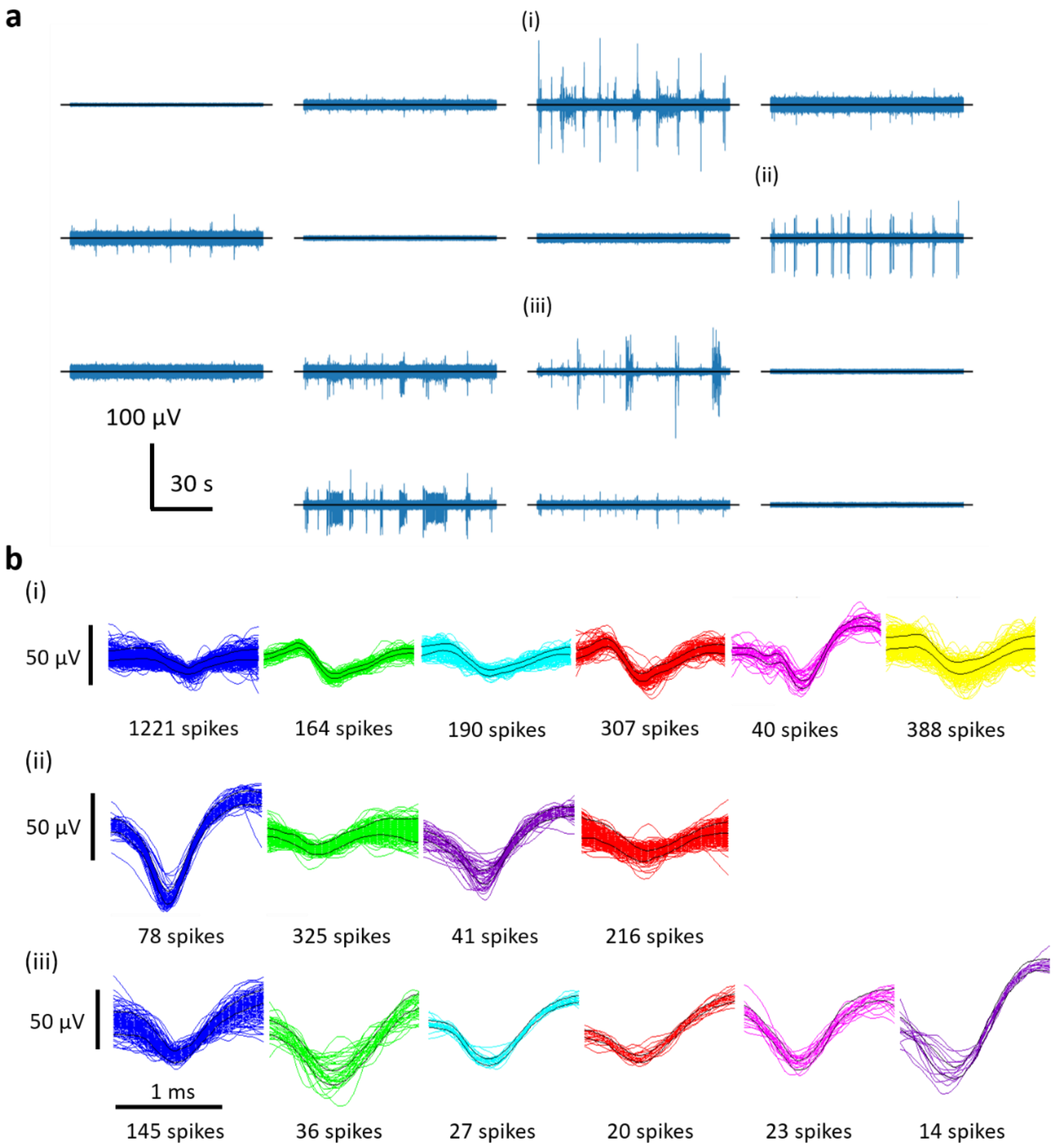

Figure S6. (a) High pass filtered recording traces showing spontaneous activity from adjacent electrodes of the transparent MEA. (b) Classification of spikes obtained from the marked recordings according to their shape, by template matching. Different groups are shown in different colors. The black outline represents the boundary of a spike template which is overlaid with individual spike waveforms. Only the major spike groups are shown here.

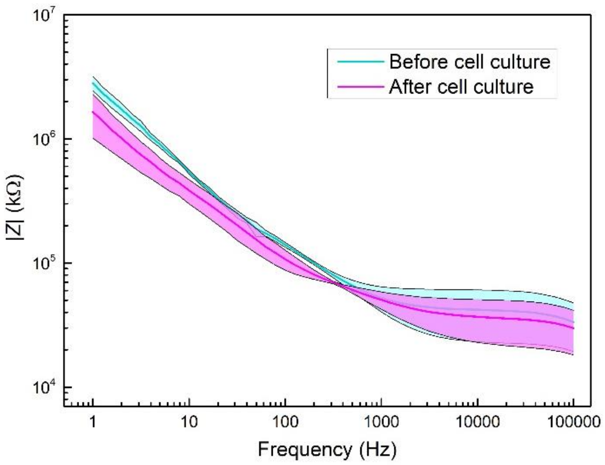

Figure S7. Comparison of electrochemical impedance spectra (magnitude) of PEDOT:PSS electrodes (n = 3) before and after 21 days of cell culture. Here, the bold lines represent the mean, while the shaded area depicts the SD.

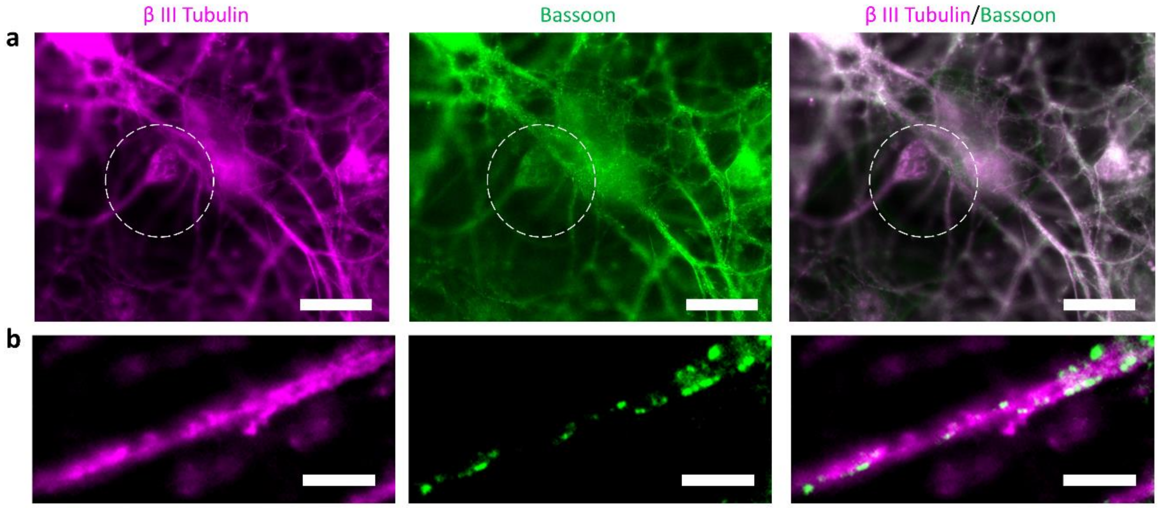

Figure S8. (a) Widefield images of neurons fixed on the MEA and stained for β-III-tubulin (magenta, left panel), bassoon protein (green, middle panel), and the overlaid image. The circular electrode is highlighted. Scale: 20 µm. (b) Magnified widefield images showing an isolated neural process located on the electrode and stained similarly as in (a). Scale: 5 µm.

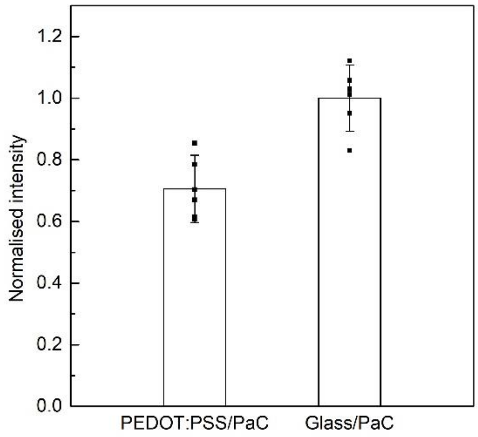

Figure S9. Comparison of the average fluorescence intensities of β-III-tubulin (red) on parylene C insulated PEDOT:PSS electrode (PEDOT:PSS/PaC) or glass (glass/PaC) regions highlighted in Figure 4c.

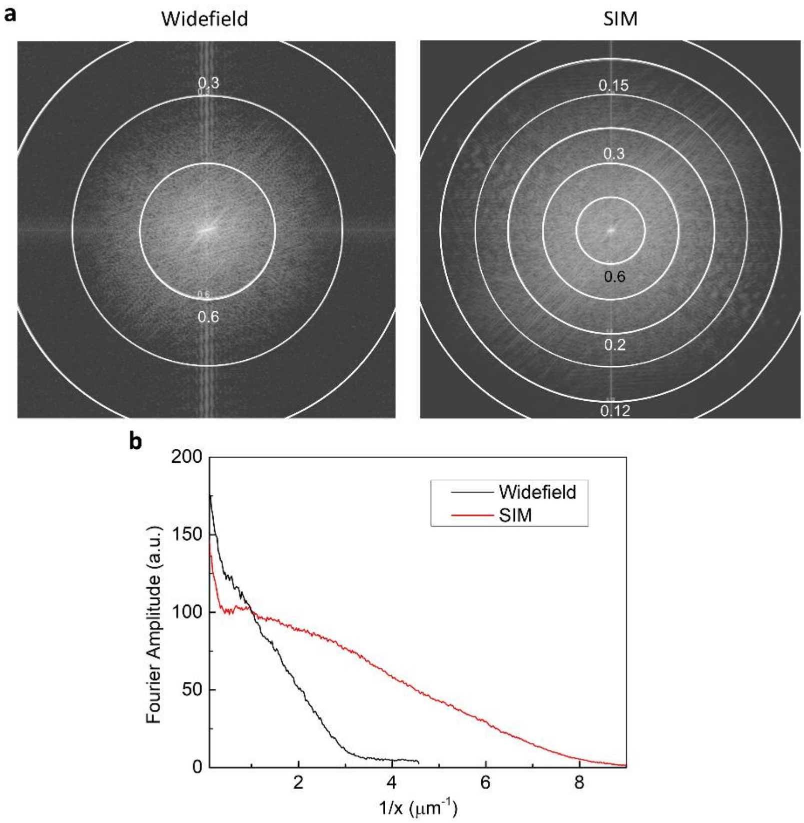

Figure S10. (a) 2D FFTs of widefield (left) and SIM images (right) of the Bassoon biomarker in Figure 5a. The concentric rings denote the feature sizes (in μm) of the image

corresponding to the spatial frequencies. (b) Averaged radial profile plots of the FFTs in (a). It illustrates higher spatial frequency information for the SIM image, as expected.

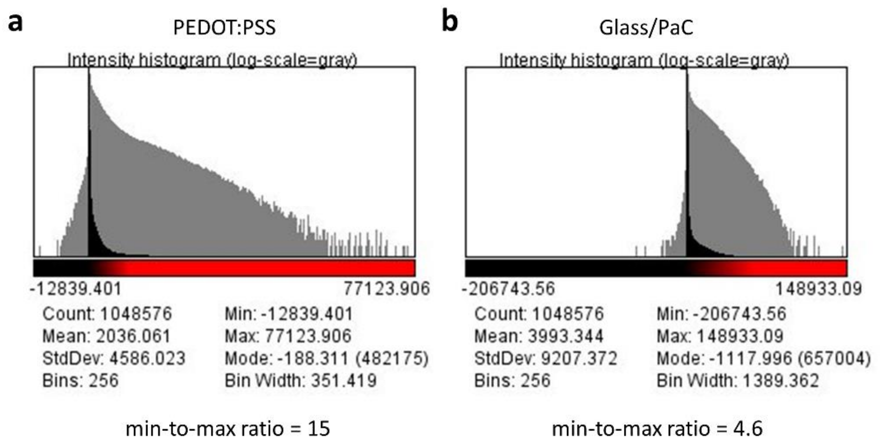

Figure S11. Pixel intensity histograms of the reconstructed SIM images of microtubule network on (a) PEDOT:PSS/PaC and (b) glass/PaC, shown in Figure 4b.

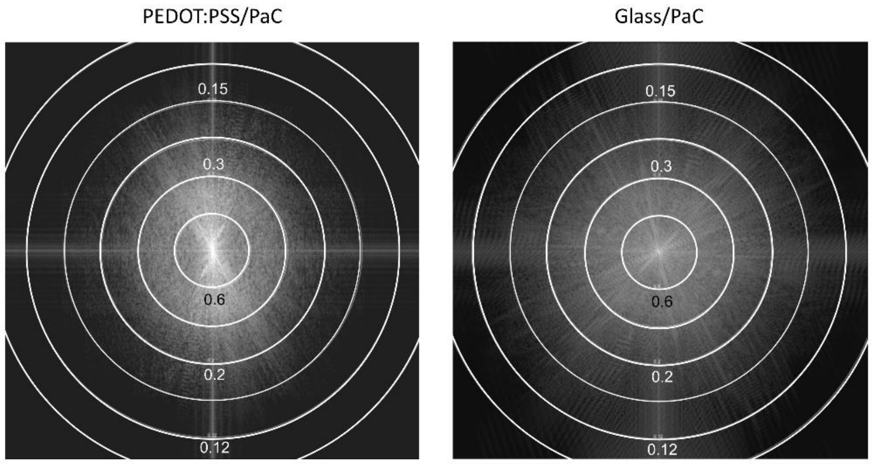

Figure S12. Greyscale representation of the false colored 2D FFTs in Figure 5c.

References

[1] K. Y. Kwon, B. Sirowatka, W. Li, A. Weber, In *2012 IEEE Biomedical Circuits and Systems Conference (BioCAS)*, **2012**, pp. 164–167.

[2] A. Zátonyi, Z. Borhegyi, M. Srivastava, D. Cserpán, Z. Somogyvári, Z. Kisvárday, Z. Fekete, *Sensors and Actuators, B: Chemical* **2018**, *273*, 519.

[3] D. Kuzum, H. Takano, E. Shim, J. C. Reed, H. Juul, A. G. Richardson, J. De Vries, H. Bink, M. A. Dichter, T. H. Lucas, D. A. Coulter, E. Cubukcu, B. Litt, *Nature Communications* **2014**, *5*, 1.

[4] D. W. Park, A. A. Schendel, S. Mikael, S. K. Brodnick, T. J. Richner, J. P. Ness, M. R. Hayat, F. Atry, S. T. Frye, R. Pashaie, S. Thongpang, Z. Ma, J. C. Williams, *Nature Communications* **2014**, *5*, 1.

[5] K. J. Seo, Y. Qiang, I. Bilgin, S. Kar, C. Vinegoni, R. Weissleder, H. Fang, *ACS Nano* **2017**, *11*, 4365.

[6] Y. Qiang, P. Artoni, K. J. Seo, S. Culaclii, V. Hogan, X. Zhao, Y. Zhong, X. Han, P. M. Wang, Y. K. Lo, Y. Li, H. A. Patel, Y. Huang, A. Sambangi, J. S. V. Chu, W. Liu, M. Fagiolini, H. Fang, *Science Advances* **2018**, *4*.

[7] P. Kshirsagar, S. Dickreuter, M. Mierzejewski, C. J. Burkhardt, T. Chassé, M. Fleischer, P. D. Jones, *Advanced Materials Technologies* **2019**, *4*, 1.

[8] M. Sessolo, D. Khodagholy, J. Rivnay, F. Maddalena, M. Gleyzes, E. Steidl, B. Buisson, G. G. Malliaras, *Advanced Materials* **2013**, *25*, 2135.
